# Immature/total granulocyte ratio improves early prediction of neurological outcome after out-of-hospital cardiac arrest: the MyeloScore study

**DOI:** 10.1186/s13613-016-0170-4

**Published:** 2016-07-16

**Authors:** Bertrand Sauneuf, Claire Bouffard, Edouard Cornet, Cedric Daubin, Jennifer Brunet, Amélie Seguin, Xavier Valette, Nicolas Chapuis, Damien du Cheyron, Jean-Jacques Parienti, Nicolas Terzi

**Affiliations:** Service de Réanimation Médicale Polyvalente, Centre Hospitalier Public du Cotentin, BP 208, 50102 Cherbourg-Octeville, France; Service de Réanimation Médicale, Centre Hospitalier Universitaire de Caen, Avenue de la Côte de Nacre, 14033 Caen, France; Laboratoire d’Hématologie, Centre Hospitalier Universitaire de Caen, Avenue de la Côte de Nacre, 14033 Caen, France; Service d’Hématologie Biologique, Hôpital Cochin, AP-HP, Paris, France; Unité de Biostatistique et de Recherche Clinique, Centre Hospitalier Universitaire de Caen, Avenue de la Côte de Nacre, 14033 Caen, France; Faculté de Médecine, EA 4652 – MILPAT, Université de Caen Basse-Normandie, 14033 Caen, France; Inserm U 1075 COMETE, 14032 Caen, France; Faculté de Médecine, EA 4655 U2RM, Université de Caen Basse-Normandie, 14032 Caen, France; Faculté de Médecine, Université de Caen Basse-Normandie, 14032 Caen, France; Institut Cochin, CNRS (UMR8104), INSERM, U1016, Université Paris Descartes, Paris, France; HP2, Inserm U1042, Université Grenoble-Alpes, 38000 Grenoble, France; Service de réanimation médicale, CHU Grenoble Alpes, 38000 Grenoble, France; Faculté de Médecine, Université Grenoble-Alpes, 38000 Grenoble, France

**Keywords:** Out-of-hospital cardiac arrest, Prognosis, Disability, Survival, Biological markers

## Abstract

**Background:**

Elevation of the immature/total granulocyte (I/T-G) ratio has been reported after out-of-hospital cardiac arrest (OHCA). Our purpose here was to evaluate the prognostic significance of the I/T-G ratio and to investigate whether the I/T-G ratio improves neurological outcome prediction after OHCA.

**Methods:**

This single-center prospective cohort study included consecutive immunocompetent patients admitted to our intensive care unit over a 3-year period (2012–2014) after successfully resuscitated OHCA. The I/T-G ratio was determined in blood samples collected at admission.

**Results:**

We studied 204 patients (77 % male, median age, 58 [48–67] years), of whom 64 % had a suspected cardiac cause of OHCA, 62 % died in the unit, and 31.5 % survived with good cerebral function. Independent outcome predictors by multivariate analysis were age, first shockable rhythm, bystander-initiated resuscitation, and I/T-G ratio. Compared to the model computed without the I/T-G ratio, the model with the ratio performed significantly better [areas under the ROC curves (AUCs), 0.78 vs. 0.83, respectively; *P* = 0.04]. These items were used to develop the MyeloScore equation: ([0.47 × I/T-G ratio] + [0.023 × age in years]) − 1.26 if initial VF/VT − 1.1 if bystander-initiated CPR. The MyeloScore predicted neurological outcomes with similar accuracy to the previously reported OHCA score (0.83 and 0.85, respectively; *P* = 0.6). The ROC–AUC was 0.84, providing external validation of the MyeloScore.

**Conclusions:**

The I/T-G ratio independently predicts neurological outcome after OHCA and, when added to other known risk factors, improves neurological outcome prediction. The clinical performance of the MyeloScore requires evaluation in a prospective study.

## Background

Out-of-hospital cardiac arrest (OHCA) is a major cause of death in Western countries with yearly estimates of 235,000–325,000 in the USA and 350,000–700,000 in Europe [1, 2]. Among patients who recover spontaneous circulation after an OHCA, very few survive with sufficient neurological function to ensure self-sufficiency [3]. Predicting the neurological outcome of patients who are comatose after an OHCA therefore has major ethical and socioeconomic implications [4] yet is an extraordinarily challenging task. Several clinical, laboratory, and electrophysiological risk factors have been identified [5]. Nevertheless, an accurate assessment of the neurological prognosis is usually possible only after several days in the intensive care unit (ICU). In most patients, the neurological prognosis cannot be assessed accurately until several days after admission to the intensive care unit (ICU).

The systemic inflammatory response syndrome (SIRS) initially described in patients with sepsis also develops after successfully resuscitated cardiac arrest. One feature of SIRS is elevation of the immature/total granulocyte (I/T-G) ratio, which is associated with mortality in both neonates and adults with sepsis [6, 7]. In a recent prospective observational study, we documented I/T-G elevation after OHCA and showed that this feature was associated with postcardiac arrest syndrome (PCAS) and ICU mortality [8].

The main objective of this single-center prospective cohort study was to evaluate the performance of I/T-G in predicting neurological outcomes after OHCA. The secondary objective was to develop a score for improving the early accurate prediction of neurological outcomes.

## Methods

The study was approved by the our institutional review board (*Comité de Protection des Personnes Nord*-*Ouest*), which waived the requirement for written informed consent, as all study procedures were either components of standard care or involved minimal risk to the patients. Nevertheless, oral informed consent was obtained from each patient or next of kin.

### Patients

We prospectively included consecutive adults (>18 years) admitted to the medical ICU of the University Hospital, in Caen, France, between June 2012 and November 2014, with successfully resuscitated OHCA. Patients were included if they presented a stable return to spontaneous circulation (ROSC). Some of these patients were also included in a previously published study (130/204 patients) [8]. We excluded pregnant women and patients with immunosuppression (hematological malignancies, solid-organ or bone marrow transplant recipients, leukocyte count <1 × 10^9^/L, corticosteroids in a daily dosage >0.5 mg/kg of prednisone-equivalent, or other immunosuppressants), and patients with refractory cardiac arrest.

For external validation, we used patients with OHCA admitted to the Cochin University Hospital in Paris, France, between January 1, 2014, and December 31, 2015.

### Care of patients with OHCA

All patients with a sustained return to spontaneous circulation (ROSC) after OHCA were admitted to our ICU for management according to our standard protocol, which included close monitoring, hemodynamic support, mechanical ventilation, and analgesia/sedation. Briefly, emergency coronary angiography was performed whenever a cardiac etiology could not be ruled out. Therapeutic hypothermia (TH) was used in all patients with initial ventricular fibrillation (VF) or ventricular tachycardia (VT) [9] and in those patients with initial pulseless electric activity (PEA) or asystole who had a suspected cardiac etiology or were expected by the physicians to have good outcomes. TH with a target temperature of 33–34 °C was induced then maintained for 12–24 h using an endovascular cooling catheter (Icy™ Catheter; Alsius, Irvine, CA, USA). During TH, the patients received adjusted midazolam and sufentanil dosages for sedation, combined with atracurium. Sedation was stopped at TH discontinuation in patients without shock or other complications or, in patients not managed with TH, at ICU admission. After TH, the target rewarming rate was 0.5 °C/h and the target temperature was 37 °C.

Neurological function was assessed by daily clinical neurological examinations and, between day 2 and day 4, by serum neuron-specific enolase (NSE) measurements, electroencephalography (EEG), and cortical somatosensory evoked potential (SSEP) recordings. Electrophysiological tests were performed without sedation and, in patients who received TH, after rewarming. In patients with bilateral absence of cortical SSEPs (N20) [10], further treatment was considered futile, and active care was withdrawn. In addition, in accordance with previous reports [5, 11, 12], for patients with at least three adverse prognostic factors after 72 h, withdrawal of active care was discussed at a staff meeting held 5 days after ICU admission. These adverse prognostic factors were absence of pupillary light reflexes or corneal reflexes, extensor posturing or no motor responses to painful stimulation, persistent myoclonus [13], serum NSE elevation [14], and a malignant EEG pattern [15].

### Immature granulocyte counts

Each included patient had a blood sample drawn at ICU admission for blood cell counts and automated analysis of leukocyte differentials using the XE-2100 system (Sysmex, Kobe, Japan). The immature granulocyte population was composed of promyelocytes, myelocytes, and metamyelocytes, but not blasts. As described elsewhere [16], a lysing reagent was used to lyse the erythrocytes and to create ultramicroscopic pores in the leukocyte cell membranes that allowed the entry of a polymethine dye with high affinity for nucleic acids. The leukocytes were then analyzed based on nucleic-acid fluorescence and side scatter [17].

### Data collection

The following data were collected prospectively for each patient, according to the Utstein style [18]: demographics, cause of OHCA, initial heart rhythm, time from collapse to basic life support (no-flow time) and from basic life support to ROSC (low-flow time), Simplified Acute Physiology Score II (SAPS II) [19] and Sequential Organ Failure Assessment (SOFA) score [20] on day 1, laboratory findings at ICU admission, ICU mortality, and cause of death. The OHCA score was calculated for each patient as follows [21]: [−13 if the initial recorded rhythm is VF or VT] + [6 × ln(no-flow time)] + [9 × ln(low-flow time)] − [1434/(serum creatinine)] + [10 × ln(arterial lactate)]. The CAHP score was calculated based on the previously published nomogram [22]. Postresuscitation shock was defined as a need for intravenous vasoactive therapy (epinephrine or norepinephrine) for more than 6 h after ROSC despite adequate fluid loading.

### Neurological outcome assessment

The primary evaluation criterion was the cerebral performance category (CPC) at hospital discharge. For this study, we defined a good neurological outcome as CPC 1 or 2, that is, alive with good cerebral performance (CPC 1) or sufficient cerebral function for independent activities of daily living, with or without mild neurological or psychological deficits (CPC 2). CPC scores 3–5, that is, severe cerebral disability (CPC 3), coma or vegetative state (CPC 4), and death (CPC 5), were defined as a poor neurological outcome.

### Statistical analysis

Quantitative variables were described as mean and standard deviation if normally distributed and as median [25th–75th percentiles] otherwise. Qualitative variables were described as *n* (%). To compare the two groups (good [CPC 1–2] and poor [CPC 3–5] neurological outcome at hospital discharge), we used Student’s *t* test for means, the Mann–Whitney *U* test for medians, and the Chi-square test (or Fisher’s exact test when the sample was small) for percentages.

We first performed a univariate analysis to identify patient characteristics at ICU admission that were associated with a good neurological outcome. These variables were then selected in a multivariate logistic regression via a stepwise procedure to build a multivariate model designed to identify factors independently and significantly associated with good neurological outcome. The odds ratios (ORs) and adjusted ORs (aORs) were computed with their 95 % confidence intervals (95 % CIs).

Maximum-likelihood estimation was then performed to analyze all factors available on ICU admission and significantly associated with the neurological outcome in the multivariate model, and the results were used to develop a predictive score (the MyeloScore). The receiver operating characteristics (ROC) curve of this score was plotted and the area under the curve (AUC–ROC) computed (c-index) to assess performance in predicting the neurological outcome.

We used SAS version 15.0 (Chicago, IL, USA) for data analysis. All tests were two-sided, and *P* values <0.05 were considered statistically significant.

## Results

### Patient characteristics

**Fig. 1:**
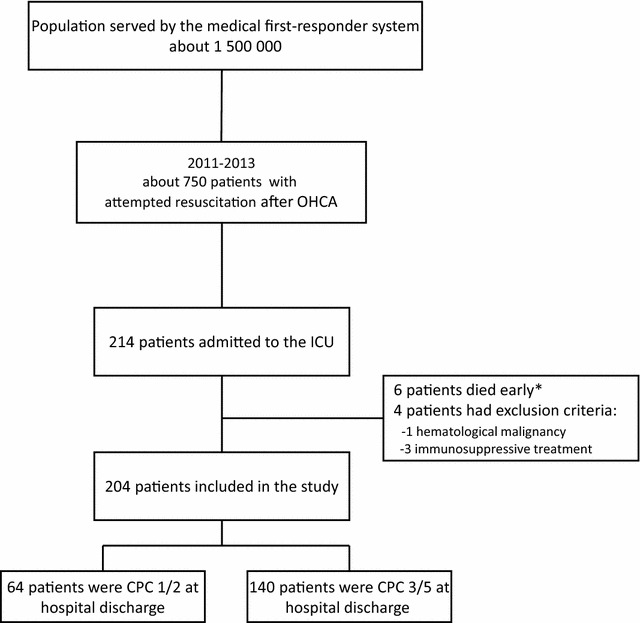
Study flowchart. *6 died early after admission, 4 of them had cardiac arrest recurrence, and 2 of them presented a refractory postcardiac arrest shock (*n* = 2). All of them died at the admission to ICU, before blood samples were drawn. These patients were not included in the analysis

Of the 214 OHCA survivors admitted to our ICU during the 3-year study period, 6 died early after admission, 4 of them had cardiac arrest recurrence, and 2 of them presented a refractory postcardiac arrest shock (*n* = 2). All of them died at the admission to ICU, before blood samples were drawn. Four had immune deficiencies (immunosuppressants in 3 and hematological malignancy in 1). These patients were not included in the analysis. Thus, 204 patients were studied (Fig. 1). Their main characteristics are listed in Table 1.

**Table 1:** Baseline characteristics of the patients included in the development cohort

Variable	All patients (*n* = 204)
*Patient demographics*	
Age (years)	58 [48–67]
Male sex, *n* (%)	156 (76.5)
Body mass index (kg/m^2^)	26 [23–28]
Charlson comorbidity index	0 [0–1]
*OHCA characteristics*	
Location of OHCA, *n* (%)	
Public place	65 (32)
Private place	139 (68)
Witnessed OHCA, *n* (%)	168 (82.5)
Bystander CPR, *n* (%)	110 (54)
Arrest rhythm, *n* (%)	
Initial VF/VT	91 (44.6)
PEA/asystole	113 (55.5)
No-flow time (min)	4 [0–10]
Low-flow time (min)	20 [15–30]
On-scene epinephrine dose (mg)	2 [1–5]
*Laboratory values at ICU admission*	
Arterial lactate (mmol/L)	4 [2–7.5]
Creatinine (µmol/L)	103 [84–142]
Troponin (µg/L)	1 [0–11]
Leukocytes (g/L)	15 [11–19.5]
Neutrophils (g/L)	12 [8–16]
I/T-G ratio (%)	0.9 [0.5–2]
*Severity scores*	
SOFA on day 1	11 [8–13]
SAPS II on day 1	69.5 [59–80]
OHCA score	29.5 [6.5–43.5]
CAHP score	170 [142–200]
*Hospital data*	
Admission temperature (°C)	35 [34–35.5]
Therapeutic hypothermia, *n* (%)	130 (63)
Early coronary angioplasty, *n* (%)	62 (30.5)
Postresuscitation shock, *n* (%)	107 (52.5)
Hospital length of stay (days)	8 [3–17]

Data are expressed in median [IQR] and *n* (%)

No-flow time is the time between collapse and base life support. Low-flow time is the time between base life support and return of spontaneous circulation

*CPC* cerebral performance category *OHCA* out-of-hospital cardiac arrest, *CPR* cardiopulmonary resuscitation, *VF/VT* ventricular fibrillation/ventricular tachycardia, *PEA* pulseless electrical activity, *I/T*-*G* ratio of immature over total granulocytes in peripheral blood, *SOFA* Sequential Organ Failure Assessment, *SAPS II*, Simplified Acute Physiology Score version II

The patients were predominantly male (*n* = 156, 77 %). A cardiac cause was identified in 130 (64 %) patients. Noncardiac causes were respiratory (*n* = 26), hanging (*n* = 18), neurological (*n* = 6), and miscellaneous (*n* = 24). Coronary angiography was performed in 131 (64 %) patients, and angioplasty in 62 (30 %). TH was induced in 85/91 (93 %) patients with VF/VT and 45/113 (40 %) patients with PEA/asystole. The ICU mortality rate was 62 % (126 patients).

### Risk factors associated with a poor neurological outcome (CPC 3–5)

Of the 204 patients, 64 (31.5 %) were CPC 1 or 2 at hospital discharge. Table 2 reports the variables significantly associated with a good neurological outcome by univariate analysis. Those available at ICU admission were younger age, cardiac arrest in a public place, witnessed arrest, bystander-initiated CPR, and on-scene epinephrine therapy. Laboratory variables associated with a good neurological outcome were lower values for arterial lactate, creatinine, and I/T-G. The ROC–AUC for hospital neurological outcome prediction of I/T-G ratio was 0.75 [0.68–0.82].

**Table 2:** Univariate analysis of factors associated with outcome at hospital discharge after out-of-hospital cardiac arrest

Variable	CPC 3–5 (*n* = 140)	CPC 1–2 (*n* = 64)	*P*
*Patient demographics*			
Age (years)	61 [48–69]	53 [46–62]	0.02
Male sex, *n* (%)	102 (73)	54 (84.5)	0.14
Body mass index (kg/m^2^)	25.5 [22–29.5]	26.4 [23.1–28]	0.71
Charlson comorbidity index	1 [0–1]	0 [0–1]	0.31
*OHCA characteristics*			
Location of OHCA, *n* (%)			0.005
Public place	36 (26)	29 (45.5)	
Private place	104 (74)	35 (54.5)	
Witnessed OHCA, *n* (%)	109 (78)	59 (92)	0.02
Bystander CPR, *n* (%)	60 (43)	50 (78)	<0.001
Arrest rhythm, *n* (%)			<0.001
Initial VF/VT	47 (33.5)	44 (69)	
PEA/asystole	93 (66.5)	20 (31)	
No-flow time (min)	6 [1–10.5]	0 [0–1]	<0.001
Low-flow time (min)	23 [15–30.5]	16 [10–25]	0.003
On-scene epinephrine dose (mg)	3 [1.5–5]	1 [0–3]	<0.001
*Laboratory values at ICU admission*			
Arterial lactate (mmol/L)	5 [2.5–8.4]	2 [1.5–4.3]	<0.001
Creatinine (µmol/L)	113 [90–149]	92 [75–111]	<0.001
Troponin (µg/L)	0.8 [0.1–8]	1.7 [0.3–16]	0.07
Leukocytes (g/L)	14.5 [11–19.5]	15 [11.5–20]	0.53
Neutrophils (g/L)	11.5 [8–15]	13 [9–17]	0.15
I/T-G ratio (%)	1.5 [0.5–2.5]	0.5 [0.3–0.9]	<0.001
*Severity scores*			
SOFA on day 1	11 [8.5–14]	9.5 [7–11]	0.001
SAPS II on day 1	74 [61–85]	63.5 [50–72]	<0.001
OHCA score	38.5 [22–46]	5 [–3–18	<0.001
CAHP score	184.5 [156–210]	144 [113–165]	<0.001
*Hospital data*			
Admission temperature (°C)	35 [34–35.5]	35 [34.5–36]	0.20
Therapeutic hypothermia, *n* (%)	75 (53.5)	55 (86)	<0.001
Early coronary angioplasty, *n* (%)	29 (20.5)	33 (51.5)	0.003
Postresuscitation shock, *n* (%)	79 (56.5)	28 (44)	0.09
Hospital length of stay (days)	5 [3–9]	16 [12–21]	<0.001

Data are expressed in median [IQR] and *n* (%)

No-flow time is the time between collapse and base life support. Low-flow time is the time between base life support and return of spontaneous circulation

*CPC* cerebral performance category, *OHCA* out-of-hospital cardiac arrest, *CPR* cardiopulmonary resuscitation, *VF/VT* ventricular fibrillation/ventricular tachycardia, *PEA* pulseless electrical activity, *I/T*-*G* ratio of immature over total granulocytes in peripheral blood, *SOFA* Sequential Organ Failure Assessment, *SAPS II* Simplified Acute Physiology Score version II

Variables independently associated with a good neurological outcome by multivariable logistic regression were younger age, initial VF/VT, bystander-initiated cardiopulmonary resuscitation (CPR), and lower I/T-G ratio (Table 3).

**Table 3:** Multivariable analysis: independent predictors of outcome at hospital discharge after out-of-hospital cardiac arrest

Variables at ICU admission	OR	95 % CI	*P* value
Age (per year)	0.98	[0.95–1]	0.06
VF/VT as initial rhythm	3.5	[1.7–7.4]	<0.001
Bystander-initiated CPR	3	[1.4–6.4]	0.005
I/T-G ratio (per 0.1)	0.62	[0.45–0.89]	0.006

c-index of the model, 0.83; goodness-of-fit (Hosmer–Lemeshow) Chi-square *P* value, 0.18

*ICU* intensive care unit, *OR* odds ratio, *95* *% CI* 95 % confidence interval, *VF/VT* ventricular fibrillation/ventricular tachycardia, *CPR* cardiopulmonary resuscitation, *I/T*-*G* ratio of immature over total granulocytes in peripheral blood

### Performance of the MyeloScore

#### MyeloScore development

The equation for computing the MyeloScore based on variables available at ICU admission and independently associated with the neurological outcome at hospital discharge was as follows:

$$\left( {\left[ {0.47 \times {\text{I}}/{\text{T-G}}\;{\text{ratio}}} \right] + \left[ {0.023 \times {\text{Age}}\;{\text{in}}\;{\text{years}}} \right]} \right) - 1.26\;{\text{if}}\;{\text{initial}}\;{\text{VF}}/{\text{VT}} - 1.1\;{\text{if}}\;{\text{bystander - initiated}}\;{\text{CPR}}$$

**Fig. 2:**
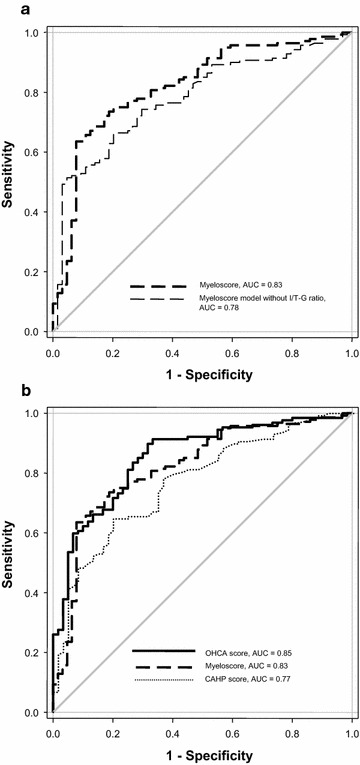
**a** Receiver operating characteristics curves for MyeloScore model with and without I/T-G ratio. **b** Receiver operating characteristics curves for MyeloScore, OHCA score, and CAHP score calculated at ICU admission

As shown in Fig. 2a, the ROC–AUC of the multivariate model described above performed significantly better than the same model without I/T-G in predicting the neurological outcome at hospital discharge (ROC–AUC, 0.83 vs. 0.78; *P* = 0.04). Thus, adding I/T-G to established risk factors improved the ability to discriminate between patients with and without good neurological outcomes.

The MyeloScore performed similarly to the previously reported OHCA score in terms of predicting a good neurological outcome (CPC 1 or 2) at hospital discharge [21]. However, a significant difference was noted between the MyeloScore and the CAHP score (ROC–AUC, 0.83 vs. 0.77; *P* = 0.02) (Fig. 2b).

The optimal cutoff value determined using the Yuden index was 1.044, and the sensitivity and specificity of the MyeloScore were 62.8 and 92.2 %, respectively.

#### MyeloScore validation

During the 2-year study period, 253 patients with OHCA were admitted to the medical ICU of the Cochin University Hospital. The I/T-G ratio at admission was available for 216 of these patients, who served for a validation study (Table 4). The ROC curve was plotted to assess the ability of the MyeloScore to separate patients with and without good neurological outcomes. The ROC–AUC was 0.84 [0.79–0.89], indicating good performance.

**Table 4:** Baseline characteristics of the 216 patients included in the validation cohort (MyeloScore variables)

Variables at ICU admission	All patients (*n* = 216)
Age (years)	65.9 [55.4–76.5]
VF/VT as initial rhythm	115 (53)
Bystander-initiated CPR, *n* (%)	144 (66.7)
I/T-G ratio (%)	1.4 [0.8–3]

*ICU* intensive care unit, *OR* odds ratio, *95* *% CI* 95 % confidence interval, *VF/VT* ventricular fibrillation/ventricular tachycardia, *CPR* cardiopulmonary resuscitation, *I/T*-*G* ratio of immature over total granulocytes in peripheral blood

## Discussion

The identification of biomarkers that predict the neurological outcome of successfully resuscitated OHCA is an area of active investigation. A few such biomarkers have been shown to perform well and are now used routinely, although they are helpful only several days after ROSC. We have reported that a high I/T-G ratio at ICU admission predicted ICU mortality after OHCA [8], and another group has confirmed this finding [23]. Here, in a large prospective cohort of patients after OHCA, we demonstrated that a higher I/T-G ratio at ICU admission independently predicted severe disability or death (CPC 3–5). Moreover, the admission I/T-G ratio provided prognostic information over and above that given by three previously established risk factors, namely age, VF/VT as the initial recorded rhythm, and bystander-initiated CPR. The MyeloScore based on all four factors performed well in discriminating between patients who would and would not survive with good neurological function. All the factors used in the MyeloScore are available at ICU admission.

I/T-G elevation, or granulocyte left shift, indicates an increase in circulating immature granulocytes and occurs in various acute conditions such as acute hematological malignancies, bleeding, and sepsis. An increase in circulating counts of immature granulocytes is a criterion for SIRS [24]. In survivors of cardiac arrest, ischemia–reperfusion induces a systemic inflammatory process akin to SIRS [25]. This early inflammatory response is often accompanied with a combination of circulatory dysfunction and anoxic brain injury known as postcardiac arrest syndrome (PCAS) [3]. Immature neutrophils enter the circulation early in the course of PCAS. The release by the bone marrow of immature granulocytes into the circulation may involve a variety of mechanisms such as bone marrow ischemia [26], neutrophil paralysis [27], and endotoxemia [28]. The finding of I/T-G elevation at ICU admission after OHCA argues against a role for infection. Furthermore, in our previous study [8], I/T-G was not significantly associated with mean arterial pressure at admission, fluid loading during resuscitation, or body temperature at admission. Thus, I/T-G elevation after OHCA seems to reflect noninfectious systemic inflammation.

Most current blood analyzers provide the count of immature granulocytes or a similar parameter routinely, without additional cost. We chose a widely used automated blood analyzer (XE 2100) to evaluate blood cell counts and I/T-G. Studies have established that automated counting is better than manual morphology-based counting for evaluating numbers of immature granulocytes in peripheral blood [29]. Another group used the ADVIA 2120 analyzer (Siemens, Forchheim, Germany) to determine another marker for granulocyte immaturity, known as the delta neutrophil index, which was high in OHCA survivors [23]. This index was also high in patients with sepsis [6]. Thus, an increase in circulating immature granulocytes may hold prognostic value regardless of the parameter used to assess it.

The early prediction of clinical outcomes after OHCA is singularly difficult. Most of the available prognostic tools can be used only several days after ICU admission. Furthermore, the use of TH delays the evaluation of neurological function [30], increasing the need for prognostic tools that perform well early after cardiac arrest. The OHCA score is based solely on variables available at ICU admission and predicts severe neurological disability or death (CPC 3–5) [21]. It was developed and validated in several cohorts of patients in Paris, France, and then further validated in a prospective cohort study conducted in the USA [31]. The OHCA score variables are initial shockable rhythm, creatinine, arterial lactate, and durations of no-flow and low-flow intervals. These variables chiefly reflect the circumstances before and during OHCA. They are also the main determinants of PCAS severity and are independent from the quality of post-OHCA care in the ICU [32]. In our population, the OHCA score had an AUC–ROC of 0.85, indicating good performance in predicting the short-term neurological outcome.

The MyeloScore performed similarly to the OHCA score in discriminating between patients who were CPC 1–2 or CPC 3–5 at hospital discharge. Importantly, adding the I/T-G ratio to age, initial rhythm, and bystander-initiated CPR significantly improved neurological outcome prediction. Three MyeloScore items are routinely recorded by on-scene healthcare providers, and the remaining item, the I/T-G ratio, is obtained at ICU admission. The durations of the no-flow and low-flow intervals are intentionally not included in the MyeloScore, but the effects of these two variables are probably reflected by the I/T-G ratio and bystander-initiated CPR. Furthermore, data on no-flow and low-flow durations are often inaccurate, and when the arrest is unwitnessed, the no-flow interval is unknown and the OHCA cannot be computed. To minimize the impact of inaccuracies in no-flow and low-flow data, logarithmic transformations of these two variables are used, making computation of the OHCA score more complex. In addition, two other OHCA items, serum creatinine and lactate levels, can be affected not only by OHCA-related ischemia–reperfusion, but also by preexisting disease such as renal failure or cirrhosis. In our cohort, the CAHP score performed less well than the MyeloScore and OHCA score in predicting outcomes. The CAHP score was developed and validated in large cohorts but may suffer from inaccuracies related to the use of a nomogram as opposed to an equation for its computation.

Although scores can contribute to predict neurological outcomes, they provide probabilities and, therefore, cannot serve to make treatment decisions for the individual patient. Instead, neuroprognostication after OHCA should rely on a range of clinical, biological, and neurophysiological tests [33]. A poor score value considered alone cannot justify treatment limitation decisions. It can, however, be added to the many other available predictors, to help adjust the management strategy and anticipate the patient’s needs.

One limitation of our study is the single-center design, which may affect the general applicability of our findings. Furthermore, we did not assess the immune properties of the immature granulocytes. Our study merely indicates an association between a higher proportion of circulating immature granulocytes and a poorer outcome of OHCA. Whether immature granulocytes are involved in the pathogenesis of PCAS remains to be investigated. In sepsis, immature granulocytes seem to perform nearly as well as mature neutrophils in mediating innate immune functions [34]. Moreover, as in the OHCA score studies, the primary outcome in our study was the short-term CPC, at hospital discharge. Long-term CPC was not assessed. Nevertheless, in recent work, CPC at hospital discharge reflected long-term survival and seemed to hold promise for program evaluation and resuscitation research [35]. Last, we did not assess the I/T-G ratio at specific time points after admission. However, when developing the study protocol, we looked at I/T-G ratio values obtained routinely on the day after ICU admission (data not shown). At this time point, often more than 12 h after ROSC, the I/T-G ratio was consistently low (between 0.2 and 0.5 %), even in patients with poor outcomes. Thus, the I/T-G ratio determined later than in our study may not have predictive value.

## Conclusion

After OHCA, the I/T-G ratio at ICU admission is independently associated with the neurological outcome at hospital discharge. We used this ratio and three other readily available items to develop the MyeloScore, which performed well in predicting neurological outcomes. The wide availability of I/T-G determination in hospital laboratories may facilitate the use of this marker. Whether the MyeloScore can help in risk stratification and, therefore, in making treatment decisions deserves to be investigated.

## Abbreviations

AUC: area under the curve
CPC: cerebral performance category
CPR: cardiopulmonary resuscitation
EEG: electroencephalography
ICU: intensive care unit
I/T-G ratio: immature/total granulocyte ratio
NSE: neuron-specific enolase
OHCA: out-of-hospital cardiac arrest
PCAS: postcardiac arrest syndrom
PEA: pulseless electric activity
ROC: receiver operating characteristic
ROSC: return on spontaneous circulation
SIRS: systemic inflammatory response syndrom
SSEP: somatosensory evoked potential
TH: therapeutic hypothermia
VF/VT: ventricular fibrillation/ventricular tachycardia
